# Jasmonate Signaling during Arabidopsis Stamen Maturation

**DOI:** 10.1093/pcp/pcz201

**Published:** 2019-10-25

**Authors:** Ivan F Acosta, Marine Przybyl

**Affiliations:** Max Planck Institute for Plant Breeding Research, Carl-von-Linn�-Weg 10, 50829 Cologne, Germany

**Keywords:** Anther dehiscence, Auxin, Filament elongation, Jasmonate, Pollen viability, Stamen maturation

## Abstract

The last stages of stamen development, collectively called stamen maturation, encompass pollen viability, filament elongation and anther dehiscence or opening. These processes are essential for male fertility in Arabidopsis and require the function of jasmonate signaling. There is a good understanding of jasmonate synthesis, perception and transcriptional outputs in Arabidopsis stamens. In addition, the spatiotemporal localization of jasmonate signaling components at the tissue and cellular levels has started to emerge in recent years. However, the ultimate cellular functions activated by jasmonate to promote stamen maturation remain unknown. The hormones auxin and gibberellin have been proposed to control the activation of jasmonate synthesis to promote stamen maturation, although we hypothesize that this action is rather indirect. In this review, we examine these different areas, attempt to clarify some confusing aspects found in the literature and raise testable hypothesis that may help to further understand how jasmonate controls male fertility in Arabidopsis.

## Introduction

In angiosperms, the development of stamens and pistils, the flower organs bearing the male and female reproductive material, is exquisitely controlled to guarantee the success of offspring generation (Ma 2005, Gomez et�al. 2015, Erbasol Serbes et�al. 2019). Mutations in genes important for any stage of stamen or pistil development, from organ formation to maturation, through cell-specific differentiation and function, can lead to plant sterility (Sanders et�al. 1999). For example, in the model plant Arabidopsis, the jasmonate family of phytohormones is indispensable for the final stages of stamen development, as shown by mutants impaired in jasmonate biosynthesis or perception, which are male sterile without compromised female fertility (Feys et�al. 1994, Sanders et�al. 2000, Stintzi and Browse 2000). In contrast to most Arabidopsis male sterile mutants, jasmonate mutants display remarkably normal-looking stamens until late in development (Fig.�1A). Accordingly, pollen grains seem to develop normally after meiosis, undergoing two rounds of mitosis to produce the expected tricellular gametophyte composed of one vegetative cell and two sperm cells (McConn and Browse 1996). However, pollen grains lose viability after this point and are unable to germinate. Furthermore, the following two other processes fail to occur: the elongation of stamen filaments, which ensures that anthers reach the pistil stigmata for fertilization, and the opening of anthers (dehiscence), which is essential for pollen release (Fig.�1A;Sanders et�al. 2000, Stintzi and Browse 2000). We refer to these three aspects of stamen development as maturation. In this review, we examine our current understanding of jasmonate signaling during this process in Arabidopsis, including synthesis, perception, transcriptional changes, possible cellular functions and the influence of auxin and gibberellin. In addition, we identify open questions and potentially interesting research avenues on these topics.

**Fig. 1:**
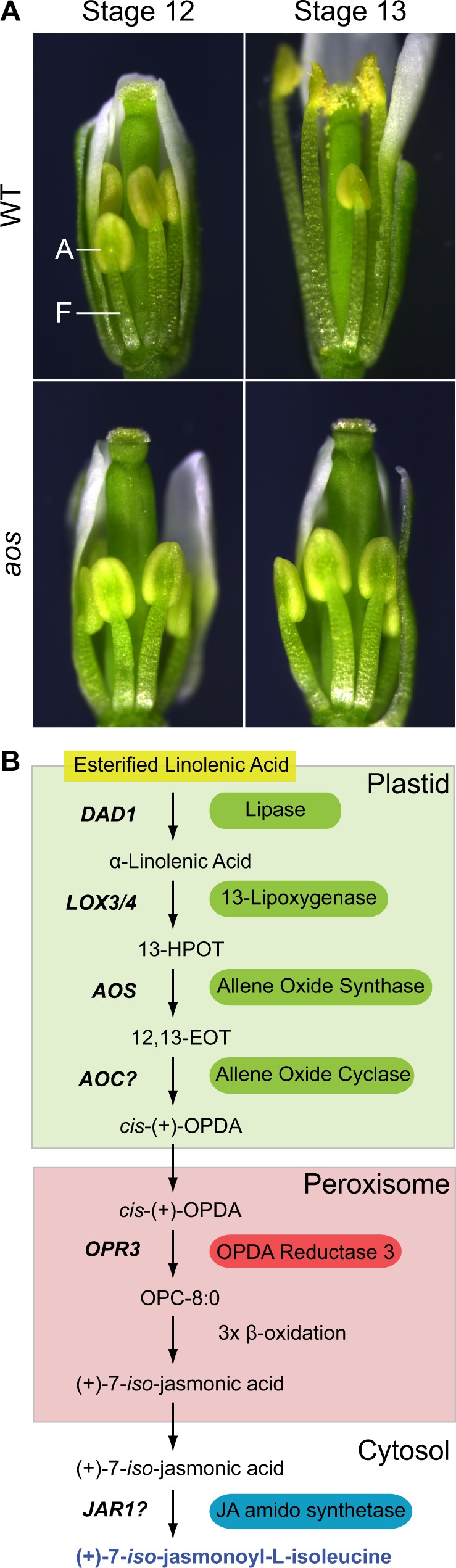
Jasmonate signaling during Arabidopsis stamen maturation. See main text for details. (A) Filament (F) elongation starts in wild-type (WT) Arabidopsis stamens at flower stage 12, and it finishes at stage 13 along with anther (A) opening. These processes fail in the jasmonate synthesis mutant *aos*. Notice that two of the six stamens in Arabidopsis flowers are always shorter and delayed. (B) Jasmonate synthesis pathway in stamens. The genes encoding the corresponding enzymes at each step are abbreviated in bold and italics. Question marks indicate that it is not yet clear which specific AOC and JAR1-type enzymes are required in stamens. 13-HPOT, 13(*S*)-hydroperoxy-octadecatrienoic acid; 12,13-EOT, (13*S*)-12,13-epoxy-octadecatrienoic acid.

## Jasmonate Synthesis

Mutant analysis of jasmonate synthesis and perception genes in Arabidopsis has provided the basis to understand several biological functions of this hormone family. The failure of stamen maturation in mutants devoid of jasmonates in Arabidopsis flowers can be rescued easily by spraying with a concentrated solution of volatile methyl jasmonate. This restores male fertility and self-pollination, allowing the propagation of pure mutant populations (Acosta and Farmer 2010). A summary of the jasmonate synthesis pathway and corresponding enzymes during Arabidopsis stamen maturation is presented in Fig.�1B.

Jasmonates are one type of oxylipins, molecules derived from the oxygenation of polyunsaturated fatty acids (Hamberg and Gardner 1992). Jasmonates in particular are made from trienoic α-linonenic acid, the most abundant polyunsaturated fatty acid in plants. One of the first hints that jasmonate is essential for Arabidopsis stamen maturation was provided by the *fad3 fad7 fad8* triple mutant. This mutant lacks α-linonenic acid due to a loss of function in all the desaturases catalyzing the last step in the synthesis of trienoic fatty acids (McConn and Browse 1996). α-Linonenic acid is mainly found within plastid membranes as part of glycerolipids, from which it is specifically released in Arabidopsis stamens by the lipase DEFECTIVE IN ANTHER DEHISCENCE1 (DAD1) to initiate jasmonate synthesis (Fig.�1B;Ishiguro et�al. 2001).

Plastid-localized 13-lipoxygenases (13-LOXs) oxygenate α-linonenic acid in carbon 13 to generate a lipid hydroperoxyde (Bannenberg et�al. 2009). The Arabidopsis genome encodes four 13-LOXs (LOX2, LOX3, LOX4 and LOX6). However, only LOX3 or LOX4 are indispensable and sufficient for stamen maturation, as demonstrated by the male sterility of the *lox3 lox4* double mutant (Caldelari et�al. 2011) and the full fertility of *lox2 lox3 lox6* and *lox2 lox4 lox6* triple mutants (Chauvin et�al. 2013). It is not yet known what determines this specific function of LOX3 and LOX4 in Arabidopsis stamens, but we can propose at least two testable explanations. First, they may be the only 13-LOXs specifically present in the relevant stamen cells or sub-plastidial compartments. Alternatively, they may be better suited than LOX2 and LOX6 to use as substrate the ‘free’ α-linonenic acid released by the DAD1 lipase.

Two subsequent enzymes, ALLENE OXIDE SYNTHASE (AOS) and ALLENE OXIDE CYCLASE (AOC), process the lipid hydroperoxyde to produce 12-oxo-phytodienoic acid (OPDA). The AOS enzyme is encoded by a single copy gene, whose loss of function abolishes all jasmonate production in the plant (Park et�al. 2002). Conversely, the AOC step is encoded by four genes, three of which are located in tandem in the Arabidopsis genome, which has so far prevented the genetic dissection of their role in stamen maturation (Stenzel et�al. 2012). OPDA is transported to peroxisomes where the synthesis of jasmonic acid is completed by an OPDA reductase (OPR3) and three rounds of β-oxidation. The vast majority of jasmonate synthesis occurs through OPR3. However, it was shown recently that OPR2, a paralog localized in the cytosol, is able to partly provide jasmonates in the *opr3* mutant through an alternative route during defense responses (Chini et�al. 2018). This route is certainly not functional in stamens, where the full sterility of the *opr3* mutant clearly indicates that OPR3 is the sole contributor to jasmonate production (Sanders et�al. 2000, Stintzi and Browse 2000).

Jasmonic acid (JA) eventually accumulates in the cytosol, where the JA-amido synthetase JASMONATE RESISTANT1 (JAR1) conjugates it to one of several amino acids (Staswick and Tiryaki 2004). By far, the most abundant bioactive jasmonate is jasmonoyl-isoleucine (JA-Ile) (Katsir et�al. 2008, Yan et�al. 2016). No JA-amido synthetase mutant devoid of JA-Ile has been identified yet, although the *jar1* mutant shows a reduction of >70% in JA-Ile levels both in flowers and after leaf wounding (Suza and Staswick 2008). Thus, another enzyme or enzymes not yet identified produce the remaining JA-Ile in these tissues. Interestingly, the *jar1* mutant is fully fertile but shows reduced jasmonate defense responses upon insect attack (Acosta et�al. 2013), suggesting that the low levels of JA-Ile in *jar1* flowers sustain stamen maturation more robustly than defense responses. Alternatively, a different jasmonate derivative or conjugate not yet known may be the bioactive molecule activating stamen maturation.

Interestingly, recent work has shown that the ABCG-type JASMONATE TRANSPORTER1 (JAT1) is important to translocate JA-Ile from the cytoplasm to the nucleus, the site of jasmonate signaling activation (Li et�al. 2017). Similar to *jar1*, the single mutant *jat1* is fertile. However, haploinsufficiency or higher-order loss of both *JAR1* and *JAT1* causes male sterility that can be rescued by methyl jasmonate application. Therefore, Li et�al. (2017) attributed this phenotype to stamen maturation defects, although a detailed description of anther, filament and pollen features is missing. These results emphasize that JA-Ile levels in *jar1* stamens are at the limit so that further reducing its transport to the nucleus abolishes jasmonate signaling. Moreover, the rescue of *jar1 jat1* fertility with methyl jasmonate supports the existence of additional unknown enzyme(s) capable of converting JA into JA-Ile in the absence of JAR1.

## Jasmonate Perception and the Activation of Transcription

Jasmonate-regulated responses are effected through large changes in gene expression that in most cases require the transcription factor MYC2, which belongs to subclade IIIe of the basic helix–loop–helix (bHLH) family (Lorenzo et�al. 2004, Fernandez-Calvo et�al. 2011). During stamen maturation, MYC2 works redundantly with its close paralogs MYC3, MYC4 and MYC5 (Qi et�al. 2015). Triple *myc* mutant combinations show delayed anther dehiscence and low pollen viability. These defects worsen in the quadruple *myc2 myc3 myc4 myc5* (*myc2/3/4/5*) mutant, which carries limited viable pollen and additionally displays slower filament elongation. Ultimately, seed set is reduced by 50% in the quadruple mutant, but this partial fertility suggests that additional jasmonate-dependent transcription factors can support the stamen maturation program. MYC2 is believed to recruit the RNA polymerase II transcriptional machinery through its interaction with MED25, a subunit of the Mediator coactivator complex (Chen et�al. 2012). However, the biological relevance of this interaction has been mainly reported for jasmonate-dependent defense responses (Kidd et�al. 2009), with no documentation of stamen maturation defects in *med25* mutants. Thus, which subunits of the Mediator complex may be required for MYC function in this process remains an open question.

The current model of jasmonate perception (Fig.�2; reviewed by Howe et�al. 2018) proposes that under low JA-Ile concentrations, a multiprotein complex coordinated by JASMONATE-ZIM-DOMAIN (JAZ) proteins represses MYC activity through several simultaneous mechanisms (Chini et�al. 2007, Thines et�al. 2007, Sheard et�al. 2010). These may include directly blocking interactions with transcriptional coactivators, or recruiting TOPLESS-type corepressors either directly or through the adaptor protein NOVEL INTERACTOR OF JAZ (NINJA; Pauwels et�al. 2010, Howe et�al. 2018). When JA-Ile accumulates, it acts as a molecular glue that brings together a coreceptor formed by JAZ proteins and CORONATINE INSENSITIVE1 (COI1). COI1 is an F-box protein that is part of an E3 ubiquitin ligase complex of the SKP1-CUL1-F-box type, which ubiquitylates JAZs, effectively sending them for degradation by the 26S proteasome (Fig.�2; Xie et�al. 1998, Thines et�al. 2007, Katsir et�al. 2008). This disassembles the entire corepressor complex, liberating MYCs to recruit the transcription machinery and activate the expression of target genes. Since a single copy gene encodes COI1 in the Arabidopsis genome, *coi1* mutants are impaired in all jasmonate-mediated responses known to date, including stamen maturation (Xie et�al. 1998). Thus, they are indistinguishable from the male sterile jasmonate synthesis mutants, except that jasmonate application obviously does not rescue *coi1* fertility. Overexpression of nondegradable JAZ proteins also causes a failure of stamen maturation, nicely supporting their role as repressors of jasmonate signaling (Thines et�al. 2007, Chung and Howe 2009).

**Fig. 2:**
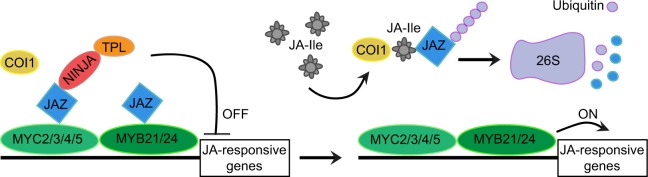
Proposed mechanism of jasmonate perception and transcriptional control of jasmonate-responsive genes with factors known to work during Arabidopsis stamen maturation. See main text for details.

The core JAZ-MYC jasmonate signaling module is a robust off/on switch that is activated by a wide range of developmental or environmental cues capable of stimulating JA-Ile accumulation (Howe et�al. 2018). On the other hand, each cue prompts specific transcriptional changes suited to the required response. Such specificity may be provided by additional transcriptional activators that interact in a combinatorial manner with MYCs (Goossens et�al. 2017, Howe et�al. 2018). For jasmonate-mediated stamen maturation, it has been proposed that the MYC partners may be at least two related R2R3-type MYB transcription factors, MYB21 and MYB24. The genes encoding these proteins were first identified as induced by jasmonate signaling at the onset of stamen maturation, along with *MYB57* and *MYB108*, two other related genes (Mandaokar et�al. 2006). Null *myb21* mutants display delayed anther opening and complete failure of filament elongation, which causes full sterility because anthers are unable to reach the pistil stigmata; however, the pollen is viable and the mutants can be propagated by manual ‘self’ pollination (Mandaokar et�al. 2006, Reeves et�al. 2012). Moreover, *myb24* mutants are completely fertile, but double *myb21 myb24* mutants fail in all three aspects of stamen maturation and are fully sterile (Mandaokar et�al. 2006, Reeves et�al. 2012). This indicates that MYB21 is alone essential for filament elongation, while it acts redundantly with MYB24 to promote pollen viability and anther dehiscence.

The earlier discovery of the *MYB21* and *MYB24* genes during stamen maturation suggested a simple scenario where the outcome of jasmonate synthesis and perception was the induced expression of these transcription factors, which would then act as ‘master regulators’ of the stamen maturation program. However, in addition to the importance of MYCs in this process, recent work uncovered that MYB21/24 physically interact with both MYC and JAZ proteins and that JAZs can inhibit their transcriptional activation function (Song et�al. 2011, Qi et�al. 2015). This suggested the slightly more complex picture of the general JAZ-MYC signaling module cooperating with MYB21/24 to specifically trigger stamen maturation after jasmonate activation (Fig.�2; Qi et�al. 2015, Goossens et�al. 2017, Howe et�al. 2018). This model supposes a pre-existing MYC-JAZ-MYB complex, but it remains an open question what controls the ‘basal’ expression of the different components. This is unclear not only for stamen maturation but also for all other jasmonate-activated responses that use similar modules throughout the plant. One possibility is that the basal transcriptional machinery constantly drives the expression of core JAZ-MYC components at low levels in all tissues, while stamen-specific factors additionally determine the basal expression of MYB21 and MYB24.

A remarkable outcome of jasmonate signaling activation is the rapid and transient accumulation of transcripts encoding jasmonate biosynthesis enzymes (e.g. *LOX2*, *LOX3* and *LOX4*), MYC transcription factors, JAZs and other repressors, and jasmonate catabolic enzymes (Chung and Howe 2009, Koo et�al. 2011). Accordingly, the expression of many of these genes is found in the transcriptome of maturing stamens (e.g. Reeves et�al. 2012). This ‘jasmonate transcriptional signature’ is believed to create positive and negative feedback loops that, on the one hand, increase the capacity to synthesize and respond to jasmonates and, on the other hand, attenuate the transcriptional signaling output (Wasternack 2007, Howe et�al. 2018). It has been suggested that positive feedback upregulation of jasmonate biosynthetic genes does not necessarily result in further jasmonate accumulation (Scholz et�al. 2015). However, mathematical modeling suggests that the two opposing loops generate a transient pulse of jasmonate biosynthesis and response (Banerjee and Bose 2011). This agrees well with the expression data of jasmonate-responsive genes in maturing stamens, where they reach a transitory expression peak at flower stage 12 that declines at stage 13 (Nagpal et�al. 2005, Reeves et�al. 2012). It is conceivable that transient pulses of jasmonate signaling are essential to correctly pattern and limit the expression and function of MYB21 and MYB24 because uncontrolled accumulation of these factors is detrimental not only to fertility but also to plant vegetative growth (Shin et�al. 2002, C. Yang et�al. 2007, Song et�al. 2011). Not surprisingly, loss of jasmonate synthesis/perception or MYC function strongly reduces the expression of the ‘jasmonate transcriptional signature’. Interestingly, however, jasmonate-responsive genes remain upregulated in the *myb21 myb24* double mutant past flower stage 13, suggesting that MYB21 and MYB24 also contribute to the negative feedback loop (Reeves et�al. 2012).

A practical aspect of the feedback upregulation of jasmonate signaling genes is that they constitute excellent markers to diagnose jasmonate signaling activation. However, it is imperative to remember that induction of a gene’s expression does not always imply an underlying function. A clear example is the jasmonate synthesis gene *LOX2*, which is highly expressed in stamens and follows the kinetics of other jasmonate-responsive genes (Reeves et�al. 2012), but it is obviously not required for the process of stamen maturation.

## Localization and Regulation of Jasmonate Synthesis and Perception in Maturing Stamens

There is limited and fragmentary information on the spatiotemporal dynamics of jasmonate synthesis factors during stamen maturation. A *DAD1* transcriptional reporter indicates that, in flowers, this gene is exclusively expressed in stamen filaments, starting shortly before the onset of stamen maturation (Ishiguro et�al. 2001). Low-resolution in situ hybridization of *OPR3* transcripts suggests a similar localization in stamen filaments and in the vascular region at the junction of anthers and filaments; however, *OPR3* expression does not seem restricted to the stamen maturation phase, being already present at early developmental stages and in additional flower organs, such as petals and pistils (Sanders et�al. 2000). Of the four *AOC* genes, only *AOC1* and *AOC4* seem active in mature stamens (filaments and pollen) according to β-glucuronidase transcriptional reporters, but an *aoc1 aoc4* double mutant did not have fertility defects (Stenzel et�al. 2012). Nevertheless, it should be noted that the T-DNA line that these authors used for *aoc4* (SALK_124897) may not be a true loss-of-function allele because their data show only marginal reductions in *AOC4* expression and the most recent update of the SALK T-DNA index indicates that this insertion lies within the 5’ untranslated region of *AOC4*. Alternatively, higher-order mutants including the other *AOC* genes may be required to unravel which of the four copies are required for stamen maturation. Lastly, immunolocalization of AOC proteins resembles the expression of *OPR3* transcripts: widespread in stamens of early flowers but restricted to filaments in maturing flowers (Hause et�al. 2003b). These authors also attempted immunocytology of AOS protein and claimed that it was found in pollen, but they did not describe the situation in filaments. In sum, filaments seem the single most shared location of jasmonate synthesis factors during stamen maturation. Although clearly more work is needed to accurately describe their spatiotemporal dynamics, an exclusive localization of jasmonate synthesis to the filaments, if confirmed, raises the interesting possibility that jasmonate exerts non-cell-autonomous effects in pollen and anther tissues.

Constitutive levels of jasmonates are normally very low in vegetative tissues but increase within seconds after mechanical wounding (Glauser et�al. 2009). In contrast, jasmonates are found at relatively high levels in flowers at the stages of stamen maturation (Reeves et�al. 2012); thus, it is expected that this accumulation is developmentally regulated. In both cases, it is not known exactly how stress or developmental signals trigger jasmonate synthesis and, for stamen maturation, there are some limited hints. Lack of the homeotic factor AGAMOUS late in stamen development causes maturation defects that can be rescued by the application of jasmonate or α-linonenic acid. Importantly, AGAMOUS seems able to bind putative *cis*-regulatory elements of the jasmonate synthesis gene *DAD1* and to ectopically activate its expression in petals (Ito et�al. 2007). Although no evidence was presented to support that AGAMOUS can induce *DAD1* in stamens, the work of Ito et�al. (2007) suggests the intriguing model that this transcription factor activates timely *DAD1* expression before the onset of stamen maturation to initiate jasmonate synthesis. In addition to this putative *direct* regulatory function of AGAMOUS, there are several reports of additional factors that seem to *indirectly* impact the initiation of jasmonate synthesis, including other hormones, such as auxin and gibberellin, that will be discussed below (Nagpal et�al. 2005, Cheng et�al. 2009, Tabata et�al. 2010, Cecchetti et�al. 2013, Peng et�al. 2013). Interestingly, most of these reports suggest *DAD1* expression as the ‘limiting’ step for jasmonate synthesis in stamens.

Recent work has shed light on the sites of jasmonate perception in maturing stamens by expressing a COI1-YFP reporter under the control of tissue- or organ-specific promoters in a *coi1-1* mutant background (Jewell and Browse 2016). First, the promoter of *COI1* rescues all three aspects of stamen maturation and confers expression in most stamen cells except pollen. This expression pattern suggests that (i) jasmonate perception is not required in pollen and is sufficient in sporophytic tissues to drive pollen viability and (ii) most stamen cells are poised for jasmonate perception and *COI1* transcription is not a limiting factor. Second, expressing COI1 only in the filament using the *DAD1* promoter partly rescues filament elongation but not anther opening or pollen viability; conversely, expressing COI1 only in anther tissues partly rescues anther opening and pollen viability but not filament elongation. These results indicate that jasmonate perception within each tissue type (anther or filament) is necessary and (only) sufficient to activate maturation within such tissue. This also emphasizes that if jasmonate production does occur only in filaments, some jasmonate should be transported to anthers to activate responses there. Third, expressing COI1 in all stamen epidermal cells suffices to partly rescue all three aspects of stamen maturation, suggesting the intriguing idea that the epidermis is the sole site of jasmonate perception in stamens. However, since the rescue is only partial, it is possible that additional cell layers need to activate jasmonate signaling for normal stamen maturation. This may be particularly expected for filament growth, where a coordinated expansion of the epidermis, cortex and vascular cell layers is likely.

In situ hybridization of *MYB21* around stage 12 finds it expressed in filaments, most strongly in the apical region and in the junction with the anthers, including the anther vasculature tissue (Cheng et�al. 2009, Reeves et�al. 2012). This pattern agrees with the expression of *OPR3* described above and fits with the role of MYB21 in filament elongation. It also suggests that jasmonate synthesis and perception in filaments suffice to activate MYB21 function there. *MYB24* shows a similar expression pattern in the filaments but seems absent from anthers (Cheng et�al. 2009, Reeves et�al. 2012). However, this absence and the restricted expression of *MYB21* in the anther vasculature are difficult to reconcile with the role of MYB21 and MYB24 in anther opening. As discussed below, one of the proposed effects of jasmonate signaling to promote anther opening is the breakage of stomium epidermal cells, which are several cell layers beyond the vasculature. Moreover, the work of Jewell and Browse (2016) suggests that jasmonate perception occurs not only in the vasculature but also in other anther cells, such as the epidermis, where it may suffice for anther opening. Thus, similar to jasmonate synthesis factors, the spatiotemporal dynamics of MYB21 and MYB24 requires more detailed analysis to clarify where exactly it occurs to promote filament elongation and anther opening.

## Possible Cellular Events Activated by Jasmonate Signaling to Drive Stamen Maturation

In contrast to the mostly clear understanding of the jasmonate signaling components required for stamen maturation, there has been only limited research on how the transcriptional reprogramming activated by jasmonate redirects cell functions to drive pollen viability, filament elongation and anther opening. The two published transcriptomes of jasmonate signaling mutants may provide a starting point to hypothesize potential executors of the stamen maturation program: the time-course transcriptome of jasmonate-deficient *opr3* mutant stamens after jasmonate treatment (Mandaokar et�al. 2006) and the differentially expressed genes in *myb21 myb24* mutant flowers at stages 12 and 13 (Reeves et�al. 2012). For example, Mandaokar et�al. (2006) proposed that jasmonate signaling induces the synthesis of waxes that may be important for pollenkitt formation and, therefore, pollen viability. Moreover, stamen filament elongation occurs through increases in cell length (Mandaokar et�al. 2006, Cheng et�al. 2009, Reeves et�al. 2012) and the transcriptomes induced by jasmonate or MYB21/24 include cell wall-modifying enzymes that may play a role in the expansion of filament cells. However, all these hypotheses remain to be tested. What seems clear is that jasmonate promotes stamen maturation by instructing different cell types to perform particular and disparate functions, such as expansion and degeneration (see below). Thus, another unsolved question is how each cell-specific response is achieved. It is possible that MYB21/24 activate the expression of a second level of transcription factors in different cell types. For example, one of the primary targets of MYB21/MYB24 may be MYB108, which seems involved in anther dehiscence only (Mandaokar and Browse 2009, Reeves et�al. 2012).

Pollen is produced and enclosed within two pairs of anther locules or chambers (Fig.�3). Adjacent locules within a pair are kept apart by a group of cells forming a septum. In addition, adjacent locules converge at the stomium, the epidermal region where the anther actually opens for pollen release. Several processes are required for anther dehiscence (represented in Fig.�3; reviewed by Wilson et�al. 2011): (i) deposition of ligno-cellulosic thickenings at the cell walls of subepidermal endothecium cells; (ii) separation or break down of septum cells, leading to locule pair fusion; (iii) separation or break down of the specialized epidermal cells forming the stomium; and (iv) dehydration of the anther surface. It is believed that this dehydration along with the tension created by the secondary thickenings of the endothecium bends the locule outward, widening the stomium opening to allow pollen release (Keijzer 1987, Nelson et�al. 2012).

**Fig. 3:**
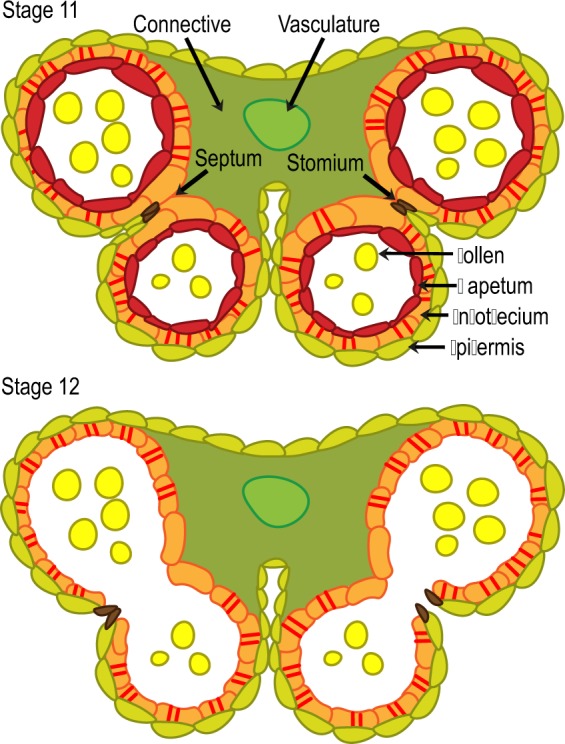
Schematic representation of Arabidopsis anther cross-sections at stages 11 and 12. Distinct cell types are shown with different colors. The tapetum layer is not visible anymore at stage 12. Red bars on the endothecium layer represent secondary thickenings. At stage 12 in this depiction, septum rupture is complete in both locule pairs, while stomium breakage to allow pollen release has only occurred in the right locule pair.

In Arabidopsis, endothecium secondary thickening does not require jasmonate signaling (Ishiguro et�al. 2001, Steiner-Lange et�al. 2003, Cecchetti et�al. 2013). Instead, it is solely dependent on the transcription factor MYB26, which partly acts by activating the expression of two other essential transcription factors, NST1 and NST2, presumed activators of genes encoding cellulose and lignin biosynthetic enzymes (Mitsuda et�al. 2005, X. Y. Yang et�al. 2007, Yang et�al. 2017). Jasmonate signaling seems only necessary for stomium breakage because anther histology shows that only this process is absent or delayed in the jasmonate-deficient mutants *dad1* and *opr3*; in contrast, they display normal septum rupture (Sanders et�al. 2000, Ishiguro et�al. 2001).

Based on several model species, it has been proposed that cell separation events in the septum and the stomium involve pectin-degrading enzymes that facilitate cell wall loosening (Keijzer 1987, Keijzer et�al. 1996, Wilson et�al. 2011). Loss-of-function mutations in a specific clade of pectin-degrading polygalacturonases of tomato (PS-2) and Arabidopsis (ADPG1, ADPG2, QRT2) block or delay anther dehiscence, supporting the importance of cell wall enzymatic lysis in this process (Gorguet et�al. 2009, Ogawa et�al. 2009). Based only on histological observations, the authors attributed this mutant defect to a failed rupture of the stomium, not the septum. The transcriptomic data suggest that jasmonate and MYB21/24 may control the expression of genes encoding these or similar pectin-degrading enzymes (Mandaokar et�al. 2006, Reeves et�al. 2012), but deeper analyses are required.

Stomium breakage has also been variously attributed to simple mechanical rupture (Keijzer 1987, Wilson et�al. 2011) or to active programmed cell death (Sanders et�al. 2000, Sanders et�al. 2005). In particular, Sanders et�al. (2000) claim that the *opr3* mutant shows a delayed anther dehiscence due to a lag in the degeneration of stomium cells, raising the interesting possibility that jasmonate activates developmentally programmed cell death. However, a clearer, unequivocal definition of the cellular and molecular events leading to the separation or break down of septum and stomium cells is still necessary. For example, it is important to detect and follow the kinetics of pectin degradation or cell death in those cells to investigate if these events are truly necessary for anther opening in Arabidopsis.

Ishiguro et�al. (2001) hypothesized that jasmonate signaling is important for anther dehydration, promoting water movement out of the anthers into the filaments, which would also cause filament cell expansion. Such mechanism would achieve an elegant synchronization of anther dehiscence and filament elongation. However, the tissue-specific rescue of jasmonate perception reviewed earlier indicates that these two processes occur mainly independent of each other (Jewell and Browse 2016). This is further supported by the phenotype of the *myb21* mutant stamens, where filaments fail to elongate but anthers open successfully although with some delay. Still, it is formally possible that water movements that follow an osmotic potential in filaments contribute to elongation (Keijzer 1987, Bonner and Dickinson 1990). Ishiguro et�al. (2001) suggested that jasmonate produced in filaments could stimulate this osmotic potential by activating the expression of the sugar transporter *AtSUC1* at the vascular interface of anther and filament tissues. Nevertheless, the transcriptome data does not support that jasmonate or MYB21/24 control the expression of *AtSUC1*. Instead, *myb21/24* mutant stamens seem to show lower expression of several ion channels that may facilitate the transport of ions, such as potassium, which could be a faster and more sensitive source of osmotic potential in filaments (Heslop-Harrison et�al. 1987, Heslop-Harrison and Heslop-Harrison 1996).

Recent work has provided genetic and physiological evidence for the requirement of dehydration in Arabidopsis anther opening and pollen viability. INDUCER OF CBF EXPRESSION 1 (ICE1), another MYC-type transcription factor, is essential for the differentiation of abaxially localized anther stomata (Wei et�al. 2018). Although stomium breakage seems to occur in an *ice1* mutant, the anther epidermis remains hydrated, preventing the widening of the stomium and, therefore, pollen release. This supports that water evaporation via stomata is essential for anther dehydration and full dehiscence and suggests that it is another event putatively activated by jasmonate signaling. Still, this does not rule out that osmotic potentials are also needed to drive additional water movements toward the filament, petals or other organs. Interestingly, jasmonate and ICE1 promote cold stress tolerance; instead, JAZ proteins repress it because they bind and block ICE1 function; thus, jasmonate likely activates ICE1 under cold stress (Hu et�al. 2013). This raises the interesting possibility that jasmonate also activates ICE1 in anthers to allow stomata differentiation. This hypothesis has not yet been tested and would imply that jasmonate signaling mutants carry undifferentiated stomata precursor cells similar to the *ice1* mutant. Alternatively, jasmonate could promote anther stomata opening as the JA-Ile mimic coronatine does in leaves during *Pseudomonas syringae* infections (Melotto et�al. 2006, Gimenez-Ibanez et�al. 2017).

## Interaction of Jasmonate Signaling with Auxin and Gibberellin

Several works have concluded that the hormones auxin and gibberellin control jasmonate synthesis through the regulation of *DAD1* expression at the onset of stamen maturation (Nagpal et�al. 2005, Cheng et�al. 2009, Tabata et�al. 2010, Reeves et�al. 2012, Cecchetti et�al. 2013). However, no *direct* effect of auxin or gibberellin signaling on the promoter of *DAD1* has been yet reported. Moreover, as detailed below, jasmonate treatments are insufficient to rescue the stamen maturation defects of mutants impaired in auxin or gibberellin signaling (Nagpal et�al. 2005, Cheng et�al. 2009). Therefore, we favor the more open interpretation that these hormones work first and foremost to complete the development of different stamen cell types, which thereby become ‘competent’ to activate *DAD1* expression and jasmonate synthesis. In this model, interrupting auxin or gibberellin signaling *indirectly* blocks jasmonate accumulation and, therefore, responses such as the jasmonate transcriptional signature. The model is summarized in Fig.�4 and detailed below.

**Fig. 4:**
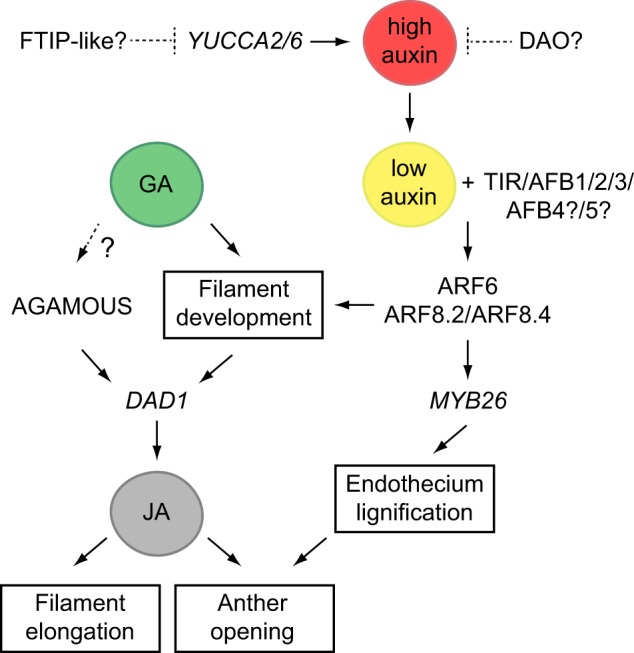
Interaction model of jasmonate with auxin and gibberellin during stamen maturation. We propose that gibberellin (GA) and auxin allow normal filament development, which is required to activate jasmonate (JA) synthesis via DAD1. Moreover, gibberellin potentially promotes AGAMOUS function, which may also induce DAD1 expression. The low specific levels of auxin required to activate ARF6/8 function might be reached through catabolism with a DAO enzyme or through downregulation of the auxin synthesis genes *YUCCA 2/6* mediated by a putative FTIP-like factor. ARF6/8 also contributes independently to anther opening by activating MYB26 expression. See main text for further details.

To investigate the relationship between gibberellin and jasmonate signaling during stamen development, Cheng et�al. (2009) used the gibberellin synthesis mutant *ga1*, which is practically devoid of gibberellins and displays pleiotropic developmental defects. In addition to having shorter stamen filament cells, *ga1* fails to produce viable pollen because microspores do not separate properly after meiosis nor undergo mitosis, and eventually degenerate. The anther wall tissue also develops abnormally and collapses (Cheng et�al. 2004). In contrast, the pollen in jasmonate mutants almost reaches maturity, arriving at the tricellular stage but losing viability afterward, and anther wall tissues are normal until late in development (Sanders et�al. 2000, Cecchetti et�al. 2013). This suggests that lack of gibberellin arrests several aspects of stamen development long before jasmonate signaling is activated. Consequently, jasmonate levels and part of the jasmonate transcriptional signature are lower in *ga1*. This correlates with an 80% reduction in the transcripts of *DAD1*, indicating that *ga1* arrested stamens are unable to initiate jasmonate synthesis (Cheng et�al. 2009). It should be noted that Cheng et�al. (2009) also considered the reduced expression of *LOX1* in *ga1* as correlative with the low jasmonate production. However, LOX1 is a 9-lipoxygenase that does not participate in jasmonate synthesis, a job performed exclusively by 13-lipoxygenases and specifically by LOX3 and LOX4 in stamens. Interestingly, treating *ga1* flowers with jasmonate does activate the expression of *MYB21* and *MYB24* (Cheng et�al. 2009), suggesting that reproductive tissues are competent to respond to jasmonate before stamen maturation, but the limiting step for signaling activation is jasmonate production. On the other hand, even if exogenous jasmonate activates signaling in the *ga1* background, this is insufficient to rescue the stamen development arrest of the mutant. This emphasizes that jasmonate synthesis is just one of several aspects that are blocked in *ga1* and that have to take place before the stamen maturation program is initiated (Cheng et�al. 2009). These authors also suggest the intriguing possibility that gibberellin signaling may activate the expression of AGAMOUS, which in turn would directly control *DAD1* expression.

All flower organs of a double mutant defective in the auxin response factors ARF6 and ARF8 arrest at stage 12, shortly before the onset of stamen maturation. Consequently, both filament elongation and anther dehiscence fail to occur (Nagpal et�al. 2005). These defects are only partial and variable in single *arf6* or *arf8* mutants indicating that these factors act in part redundantly. The arrest of *arf6 arf8* stamens seems to happen later than that of *ga1*, although no histological description has been reported. Thus, it is not clear if and when *arf6 arf8* pollen is defective, or if *arf6 arf8* anthers are affected in other processes necessary for dehiscence but independent of jasmonate signaling, such as endothecium lignification. However, class 1 *KNOX* genes, which are known as repressors of cell differentiation, are ectopically expressed in *arf6 arf8* flowers and this seems to partly account for their arrest (Tabata et�al. 2010). Moreover, reducing ARF6/8 activity appears to block filament vasculature at the procambium stage, because a procambial marker shows an expanded expression in the double mutant (Rubio-Somoza and Weigel 2013).

Similar to *ga1*, jasmonate levels in *arf6 arf8* are reduced and correlated with a lack of *DAD1* expression (Nagpal et�al. 2005, Tabata et�al. 2010). In contrast to *ga1*, jasmonate application does rescue anther opening in *arf6 arf8* but not filament elongation, so it has been concluded that the low jasmonate accumulation in this mutant is only responsible for one defect but not the other (Nagpal et�al. 2005). We propose that ARF6 and ARF8 are required for the correct development of filament cells, which are likely the source, via *DAD1* activation, of the jasmonate that triggers both filament elongation and anther opening. In this model, *arf6 arf8* filament cells remain immature, not competent to initiate the synthesis of jasmonate or to respond to it. Instead, anther tissues do mature and are obviously responsive to jasmonate but unable to synthesize it independently. In this sense, the work on ARF6 and ARF8 would support that anthers are normally not capable of triggering jasmonate synthesis, for which they are fully dependent on filaments. The potential requirement of ARF6 and ARF8 for the completion of vasculature development in the filament is remarkable, because jasmonate synthesis genes and enzymes can be expressed in vascular tissues (Hause et�al. 2003a; Gasperini et�al. 2015), which may be a suitable location for jasmonate production if transport to the anther is needed.

Recent work has shown that the function of *ARF8* in filament elongation is most likely effected through specific splice variants, mainly ARF8.4 with perhaps a minor contribution of ARF8.2 (Ghelli et�al. 2018). Inducing higher levels of *ARF8.4* in wild-type plants increases final stamen length slightly but significantly, and it restores the mild 15% reduction in filament length of the *arf8-7* single mutant. Moreover, induction of *ARF8.4* in *arf8-7* recovers the expression of *Aux/IAA19*, an auxin-responsive gene that Ghelli et al. consider a master regulator of filament elongation. However, this interpretation is inconsistent with the function of Aux/IAA19 as a repressor of auxin signaling. Analogously to JAZs in jasmonate signaling, Aux/IAA proteins repress the function of ARFs until auxin accumulation and sensing target them for degradation (Chapman and Estelle 2009). Accordingly, the dominant *MASSUGU2* mutations disrupt the degron motif of Aux/IAA19, which renders it stable and insensitive to auxin degradation (Tatematsu et�al. 2004). Thus, filament growth is delayed in *MASSUGU2* (Tashiro et�al. 2009), similar to *arf6* or *arf8* single mutants, likely because ARF6/8 remain partially repressed. Therefore, *Aux/IAA19* is a repressor of the filament cell development program enabled by ARF6 and ARF8 and has to be eliminated through auxin perception to activate ARF function. In addition, ARF6/8 probably induce the expression of not only *Aux/IAA19* but also at least five other *Aux/IAAs* [c.f. microarray data in Reeves et�al. (2012)]. Again, in analogy to jasmonate signaling, *Aux/IAA* induction probably serves a negative feedback role to limit (not stimulate) auxin-ARF output. It is also a stereotypical transcriptional response that is a practical readout of auxin-ARF signaling activation (Chapman and Estelle 2009).

Ectopically inducing higher levels of both *ARF8.2* and *ARF8.4* causes precocious anther dehiscence in wild-type and *arf8-7* anthers (Ghelli et�al. 2018). This phenomenon was correlated with an earlier endothecium lignification mediated by ARF8.4 and with an increased *DAD1* expression brought about through ARF8.2. As with any other overexpression or gain-of-function phenotype, this result shows some potential functions of ARF8.2 and ARF8.4, but this does not necessarily mean that they are actually performing these functions when expressed under their endogenous promoter. Therefore, it will be important to test if ARF8.2 is sufficient to rescue *DAD1* expression and, therefore, jasmonate biosynthesis, in the full loss-of-function mutant *arf6 arf8*. Moreover, the potential role of ARF6/8 in endothecium lignification suggests that this process is likely impaired in *arf6 arf8*. However, as mentioned earlier, this and other histological features have not been yet reported for this mutant. If such a defect is found, it will also be necessary to test if ARF8.4 suffices to drive endothecium lignification in an *arf6 arf8* double mutant.

As mentioned earlier, the canonical auxin perception mechanism is expected to activate ARF6 and ARF8. This involves degradation of Aux/IAA repressors after auxin-mediated interaction with TIR1/ABF F-box proteins. Accordingly, a *tir1 afb1 afb2 afb3* quadruple mutant shows approximately 25% reduced filament elongation with respect to the wild type (Cecchetti et�al. 2008), reminiscent of the *arf8-7* mutant. However, in stark contrast to the indehiscence of *arf6 arf8*, the *tir1 afb1 afb2 afb3* mutant shows approximately 90% precocious anther dehiscence at stage 12, when is rarely observed in the wild type (Cecchetti et�al. 2008). The triple mutant *tir1 afb2 afb3* and the single mutant *afb1* also display this phenotype at early stage 12, although at lower frequencies, 35 and 10%, respectively (Cecchetti et�al. 2013). Furthermore, pollen grains also mature prematurely in all three mutants. In agreement with these phenotypes, artificially increasing the output of auxin signaling with exogenous auxin application or ectopic expression of the Agrobacterium *rolB* oncogene can delay anther dehiscence in Arabidopsis or tobacco, respectively (Cecchetti et�al. 2004, Cecchetti et�al. 2008, Cecchetti et�al. 2013). In rice, excess accumulation of the bioactive auxin IAA blocks anther dehiscence of the *dao* mutant, impaired in an enzyme that catabolizes IAA, and of the *Osftip7* mutant, affected in a protein essential for the downregulation of the auxin synthesis gene *OsYUCCA4* (Zhao et�al. 2013, Song et�al. 2018). Collectively, this evidence supports that auxin signaling negatively regulates anther dehiscence and pollen viability. However, such conclusion seems to contradict the positive role of the auxin-dependent factors ARF6 and ARF8 in anther opening.

Similar to the ectopic expression of *ARF8.2* and *ARF8.4*, the precocious anther dehiscence in the *tir1*/*afb* mutants is due to an earlier expression of *MYB26*, which causes accelerated endothecium lignification, and to elevated jasmonate levels associated with higher *DAD1* expression (Cecchetti et�al. 2013). This deepens the contradiction because two seemingly opposite events (blocking auxin signaling and ectopically activating ARF8, an auxin-dependent factor) cause the same molecular and phenotypic effects. The root of this inconsistency may be the way Cecchetti et al. interpret the kinetics of auxin and auxin-induced DR5 reporters that they have observed in anthers (Cecchetti et�al. 2008, Cecchetti et�al. 2013). Both auxin and DR5 reporters peak after meiosis (stage 10) and then decline when endothecium lignification is complete (stage 11). Auxin concentration further decreases, and DR5 reporters are not detectable at the onset of maturation (stage 12). Moreover, DR5 reporters are inactive in *tir1*/*afb* multiple mutants. Thus, Cecchetti et�al. (2008) imply that auxin signaling in anthers is normally switched off before maturation starts to allow anther opening.

We propose instead that the reduced but substantial auxin content at stage 12 (Cecchetti et�al. 2013) does drive a lower signaling output that is specifically required to activate ARF6 and ARF8 at appropriate levels in anthers (Fig.�4). It is also possible that the different affinities of each F-box receptor for different natural auxins or auxin-related molecules may determine the specific level of signaling required for ARF6/8 function during stamen maturation. However, this degree of auxin signaling is likely insufficient to activate DR5, a promoter that does not report all auxin responses (Liao et�al. 2015, Chandler 2016). Instead, *Aux/IAA19*, another auxin response gene likely activated by ARF6 and ARF8, shows a strong, later expression (Tashiro et�al. 2009) that may reflect auxin signaling during stamen maturation more accurately. In our interpretation, auxin perception is not abolished but only reduced in the single, triple or quadruple *tir1*/*afb* mutants to a level that allows earlier ARF6/8 activation and anther opening. The partial reduction (∼25%) of filament growth in the *tir1 afb1 afb2 afb3* quadruple mutant supports this idea because it suggests that some auxin signaling is still occurring through *AFB4* and *AFB5*. These are the remaining functional *AFB* genes that probably step in to activate ARF6/8 and allow significant filament elongation. Our interpretation predicts that the precocious anther dehiscence of *tir1 afb1 afb2 afb3* requires ARF6/8 function. This can be unequivocally tested by introducing the *tir1/afb* mutations in an *arf8* or *arf6 arf8* background. Furthermore, we predict that completely removing auxin perception should not cause precocious anther dehiscence but rather prevent it entirely as in *arf6 arf8*. Recent work has generated even higher-order *tir1/afb* mutants that are viable and may allow to test this hypothesis (Prigge et�al. 2019). Finally, the inhibitory effect of high auxin (signaling) levels in anther dehiscence may be due in part to excess ARF6/8 activity, which has also been shown to negatively impact the progression of this process (Wu et�al. 2006, Zheng et�al. 2019).

## Concluding Remarks

The maturing Arabidopsis stamen has been a great model to uncover some of the important components of jasmonate signaling in general. Thus, there is clear genetic support for many of the jasmonate-related factors required for stamen maturation, including most of the specific enzymes of jasmonate synthesis, the hormone perception coreceptor and the transcription factors translating jasmonate signaling into gene expression changes. However, many interesting questions remain regarding (i) the cell-specific sites of jasmonate production and perception in stamens; (ii) the significance of the feedback expression of jasmonate-related genes; (iii) the actual cellular functions activated by gene expression to ultimately drive pollen viability, filament elongation and anther opening; and (iv) the interaction of jasmonate with other hormones during stamen maturation. We have outlined those questions and proposed ways to answer them. Continued research on the late stages of stamen development will not only refine our knowledge of jasmonate signaling but also uncover general principles for the workings of plant hormones, particularly how signaling results in specific developmental responses.

## Funding

This work was supported by core funding from the Max Planck Society to the Max Planck Institute for Plant Breeding Research.

## References

[pcz201-B1] Acosta I.F., Farmer E.E. (2010) Jasmonates. Arabidopsis Book 8: e0129. 22303255 10.1199/tab.0129 PMC3244945

[pcz201-B2] Acosta I.F., Gasperini D., Chetelat A., Stolz S., Santuari L., Farmer E.E. (2013) Role of NINJA in root jasmonate signaling. Proc. Natl. Acad. Sci. U.S.A. 110: 15473–15478. 24003128 10.1073/pnas.1307910110 PMC3780868

[pcz201-B3] Banerjee S., Bose I. (2011) Transient pulse formation in jasmonate signaling pathway. J. Theor. Biol. 273: 188–196. 21194534 10.1016/j.jtbi.2010.12.037

[pcz201-B4] Bannenberg G., Martinez M., Hamberg M., Castresana C. (2009) Diversity of the enzymatic activity in the lipoxygenase gene family of *Arabidopsis thaliana*. Lipids 44: 85–95. 18949503 10.1007/s11745-008-3245-7

[pcz201-B5] Bonner L.J., Dickinson H.G. (1990) Anther dehiscence in *Lycopersicon esculentum*. 2. Water relations. New Phytol. 115: 367–375. 10.1111/j.1469-8137.1990.tb00463.x 33873953

[pcz201-B6] Caldelari D., Wang G., Farmer E.E., Dong X. (2011) Arabidopsis lox3 lox4 double mutants are male sterile and defective in global proliferative arrest. Plant Mol. Biol. 75: 25–33. 21052784 10.1007/s11103-010-9701-9

[pcz201-B7] Cecchetti V., Altamura M.M., Brunetti P., Petrocelli V., Falasca G., Ljung K. (2013) Auxin controls Arabidopsis anther dehiscence by regulating endothecium lignification and jasmonic acid biosynthesis. Plant J. 74: 411–422. 23410518 10.1111/tpj.12130

[pcz201-B8] Cecchetti V., Altamura M.M., Falasca G., Costantino P., Cardarelli M. (2008) Auxin regulates Arabidopsis anther dehiscence, pollen maturation, and filament elongation. Plant Cell 20: 1760–1774. 18628351 10.1105/tpc.107.057570 PMC2518247

[pcz201-B9] Cecchetti V., Pomponi M., Altamura M.M., Pezzotti M., Marsilio S., D’Angeli S., et al (2004) Expression of rolB in tobacco flowers affects the coordinated processes of anther dehiscence and style elongation. Plant J 38: 512–525. 15086797 10.1111/j.0960-7412.2004.02064.x

[pcz201-B10] Chandler J.W. (2016) Auxin response factors. Plant Cell Environ. 39: 1014–1028. 26487015 10.1111/pce.12662

[pcz201-B11] Chapman E.J., Estelle M. (2009) Mechanism of auxin-regulated gene expression in plants. Annu. Rev. Genet. 43: 265–285. 19686081 10.1146/annurev-genet-102108-134148

[pcz201-B12] Chauvin A., Caldelari D., Wolfender J.L., Farmer E.E. (2013) Four 13-lipoxygenases contribute to rapid jasmonate synthesis in wounded *Arabidopsis thaliana* leaves: a role for lipoxygenase 6 in responses to long-distance wound signals. New Phytol. 197: 566–575. 23171345 10.1111/nph.12029

[pcz201-B13] Chen R., Jiang H., Li L., Zhai Q., Qi L., Zhou W., et al (2012) The Arabidopsis mediator subunit MED25 differentially regulates jasmonate and abscisic acid signaling through interacting with the MYC2 and ABI5 transcription factors. Plant Cell 24: 2898–2916. 22822206 10.1105/tpc.112.098277 PMC3426122

[pcz201-B14] Cheng H., Qin L., Lee S., Fu X., Richards D.E., Cao D., et al (2004) Gibberellin regulates Arabidopsis floral development via suppression of DELLA protein function. Development 131: 1055–1064. 14973286 10.1242/dev.00992

[pcz201-B15] Cheng H., Song S., Xiao L., Soo H.M., Cheng Z., Xie D., et al (2009) Gibberellin acts through jasmonate to control the expression of MYB21, MYB24, and MYB57 to promote stamen filament growth in Arabidopsis. PLoS Genet. 5: e1000440. 19325888 10.1371/journal.pgen.1000440 PMC2654962

[pcz201-B16] Chini A., Fonseca S., Fernandez G., Adie B., Chico J.M., Lorenzo O., et al (2007) The JAZ family of repressors is the missing link in jasmonate signalling. Nature 448: 666–671. 17637675 10.1038/nature06006

[pcz201-B17] Chini A., Monte I., Zamarreno A.M., Hamberg M., Lassueur S., Reymond P., et al (2018) An OPR3-independent pathway uses 4,5-didehydrojasmonate for jasmonate synthesis. Nat. Chem. Biol. 14: 171–178. 29291349 10.1038/nchembio.2540

[pcz201-B18] Chung H.S., Howe G.A. (2009) A critical role for the TIFY motif in repression of jasmonate signaling by a stabilized splice variant of the JASMONATE ZIM-domain protein JAZ10 in Arabidopsis. Plant Cell 21: 131–145. 19151223 10.1105/tpc.108.064097 PMC2648087

[pcz201-B19] Erbasol Serbes I., Palovaara J., Gro�-Hardt R. (2019) Development and function of the flowering plant female gametophyte. Curr. Top. Dev. Biol. 131: 401–434. 30612625 10.1016/bs.ctdb.2018.11.016

[pcz201-B20] Fernandez-Calvo P., Chini A., Fernandez-Barbero G., Chico J.M., Gimenez-Ibanez S., Geerinck J. (2011) The Arabidopsis bHLH transcription factors MYC3 and MYC4 are targets of JAZ repressors and act additively with MYC2 in the activation of jasmonate responses. Plant Cell 23: 701–715. 21335373 10.1105/tpc.110.080788 PMC3077776

[pcz201-B21] Feys B., Benedetti C.E., Penfold C.N., Turner J.G. (1994) Arabidopsis mutants selected for resistance to the phytotoxin coronatine are male sterile, insensitive to methyl jasmonate, and resistant to a bacterial pathogen. Plant Cell 6: 751–759. 12244256 10.1105/tpc.6.5.751 PMC160473

[pcz201-B22] Gasperini D., Chauvin A., Acosta I.F., Kurenda A., Stolz S., Chetelat A., et al (2015) Axial and radial oxylipin transport. Plant Physiol. 169: 2244–2254. 26338953 10.1104/pp.15.01104 PMC4634084

[pcz201-B23] Ghelli R., Brunetti P., Napoli N., De Paolis A., Cecchetti V., Tsuge T., et al (2018) A newly identified flower-specific splice variant of AUXIN RESPONSE FACTOR8 regulates stamen elongation and endothecium lignification in Arabidopsis. Plant Cell 30: 620–637. 29514943 10.1105/tpc.17.00840 PMC5894849

[pcz201-B24] Gimenez-Ibanez S., Boter M., Ortigosa A., Garcia-Casado G., Chini A., Lewsey M.G., et al (2017) JAZ2 controls stomata dynamics during bacterial invasion. New Phytol. 213: 1378–1392. 28005270 10.1111/nph.14354

[pcz201-B25] Gomez J.F., Talle B., Wilson Z.A. (2015) Anther and pollen development: a conserved developmental pathway. J. Integr. Plant Biol. 57: 876–891. 26310290 10.1111/jipb.12425 PMC4794635

[pcz201-B26] Glauser G., Dubugnon L., Mousavi S.A., Rudaz S., Wolfender J.L., Farmer E.E. (2009) Velocity estimates for signal propagation leading to systemic jasmonic acid accumulation in wounded *Arabidopsis*. J. Biol. Chem. 284: 34506–34513. 19846562 10.1074/jbc.M109.061432 PMC2787311

[pcz201-B27] Goossens J., Mertens J., Goossens A. (2017) Role and functioning of bHLH transcription factors in jasmonate signalling. J. Exp. Bot. 68: 1333–1347. 27927998 10.1093/jxb/erw440

[pcz201-B28] Gorguet B., Schipper D., van Lammeren A., Visser R.G., van Heusden A.W. (2009) ps-2, the gene responsible for functional sterility in tomato, due to non-dehiscent anthers, is the result of a mutation in a novel polygalacturonase gene. Theor. Appl. Genet. 118: 1199–1209. 19219598 10.1007/s00122-009-0974-9

[pcz201-B29] Hamberg M., Gardner H.W. (1992) Oxylipin pathway to jasmonates: biochemistry and biological significance. Biochim. Biophys. Acta 1165: 1–18. 1420338 10.1016/0005-2760(92)90069-8

[pcz201-B30] Hause B., Hause G., Kutter C., Miersch O., Wasternack C. (2003a) Enzymes of jasmonate biosynthesis occur in tomato sieve elements. Plant Cell Physiol. 44: 643–648. 12826630 10.1093/pcp/pcg072

[pcz201-B31] Hause B., Stenzel I., Miersch O., Wasternack C. (2003b) Occurrence of the allene oxide cyclase in different organs and tissues of *Arabidopsis thaliana*. Phytochemistry 64: 971–980. 14561513 10.1016/s0031-9422(03)00447-3

[pcz201-B32] Heslop-Harrison J.S., Heslop-Harrison Y., Reger B.J. (1987) Anther-filament extension in Lilium: potassium ion movement and some anatomical features. Ann. Bot. 59: 505–515.

[pcz201-B33] Heslop-Harrison Y., Heslop-Harrison J.S. (1996) Lodicule function and filament extension in the grasses: potassium ion movement and tissue specialization. Ann. Bot. 77: 573–582.

[pcz201-B34] Howe G.A., Major I.T., Koo A.J. (2018) Modularity in jasmonate signaling for multistress resilience. Annu. Rev. Plant Biol. 69: 387–415. 29539269 10.1146/annurev-arplant-042817-040047

[pcz201-B35] Hu Y., Jiang L., Wang F., Yu D. (2013) Jasmonate regulates the inducer of cbf expression-C-repeat binding factor/DRE binding factor1 cascade and freezing tolerance in Arabidopsis. Plant Cell 25: 2907–2924. 23933884 10.1105/tpc.113.112631 PMC3784588

[pcz201-B36] Ishiguro S., Kawai-Oda A., Ueda J., Nishida I., Okada K. (2001) The *DEFECTIVE IN ANTHER DEHISCIENCE1* gene encodes a novel phospholipase A1 catalyzing the initial step of jasmonic acid biosynthesis, which synchronizes pollen maturation, anther dehiscence, and flower opening in Arabidopsis. Plant Cell 13: 2191–2209. 11595796 10.1105/tpc.010192 PMC139153

[pcz201-B37] Ito T., Ng K.H., Lim T.S., Yu H., Meyerowitz E.M. (2007) The homeotic protein AGAMOUS controls late stamen development by regulating a jasmonate biosynthetic gene in *Arabidopsis*. Plant Cell 19: 3516–3529. 17981996 10.1105/tpc.107.055467 PMC2174883

[pcz201-B38] Jewell J.B., Browse J. (2016) Epidermal jasmonate perception is sufficient for all aspects of jasmonate-mediated male fertility in Arabidopsis. Plant J. 85: 634–647. 26833563 10.1111/tpj.13131

[pcz201-B39] Katsir L., Schilmiller A.L., Staswick P.E., He S.Y., Howe G.A. (2008) COI1 is a critical component of a receptor for jasmonate and the bacterial virulence factor coronatine. Proc. Natl. Acad. Sci. U.S.A. 105: 7100–7105. 18458331 10.1073/pnas.0802332105 PMC2383947

[pcz201-B40] Keijzer C.J. (1987) The processes of anther dehiscence and pollen dispersal. I. The opening mechanism of longitudinally dehiscing anthers. New Phytol. 105: 487–498. 10.1111/j.1469-8137.1987.tb00886.x 33873908

[pcz201-B41] Keijzer C.J., Leferink-Ten Klooster H.B., Reinders M.C. (1996) The mechanics of the grass flower: anther dehiscence and pollen shedding in maize. Ann. Bot. 78: 15–21.

[pcz201-B42] Kidd B.N., Edgar C.I., Kumar K.K., Aitken E.A., Schenk P.M., Manners J.M., et al (2009) The mediator complex subunit PFT1 is a key regulator of jasmonate-dependent defense in Arabidopsis. Plant Cell 21: 2237–2252. 19671879 10.1105/tpc.109.066910 PMC2751954

[pcz201-B43] Koo A.J.K., Cooke T.F., Howe G.A. (2011) Cytochrome P450 CYP94B3 mediates catabolism and inactivation of the plant hormone jasmonoyl-L-isoleucine. Proc. Natl. Acad. Sci. U.S.A. 108: 9298–9303. 21576464 10.1073/pnas.1103542108 PMC3107288

[pcz201-B44] Li Q., Zheng J., Li S., Huang G., Skilling S.J., Wang L., et al (2017) Transporter-mediated nuclear entry of jasmonoyl-isoleucine is essential for jasmonate signaling. Mol. Plant 10: 695–708. 28179150 10.1016/j.molp.2017.01.010

[pcz201-B45] Liao C.Y., Smet W., Brunoud G., Yoshida S., Vernoux T., Weijers D. (2015) Reporters for sensitive and quantitative measurement of auxin response. Nat. Methods 12: 207–210.202 p following 210. 25643149 10.1038/nmeth.3279 PMC4344836

[pcz201-B46] Lorenzo O., Chico J.M., Sanchez-Serrano J.J., Solano R. (2004) *JASMONATE-INSENSITIVE1* encodes a MYC transcription factor essential to discriminate between different jasmonate-regulated defense responses in Arabidopsis. Plant Cell 16: 1938–1950. 15208388 10.1105/tpc.022319 PMC514172

[pcz201-B47] Ma H. (2005) Molecular genetic analyses of microsporogenesis and microgametogenesis in flowering plants. Annu. Rev. Plant Biol. 56: 393–434. 15862102 10.1146/annurev.arplant.55.031903.141717

[pcz201-B48] Mandaokar A., Browse J. (2009) MYB108 acts together with MYB24 to regulate jasmonate-mediated stamen maturation in Arabidopsis. Plant Physiol. 149: 851–862. 19091873 10.1104/pp.108.132597 PMC2633834

[pcz201-B49] Mandaokar A., Thines B., Shin B., Lange B.M., Choi G., Koo Y.J., et al (2006) Transcriptional regulators of stamen development in Arabidopsis identified by transcriptional profiling. Plant J. 46: 984–1008. 16805732 10.1111/j.1365-313X.2006.02756.x

[pcz201-B50] McConn M., Browse J. (1996) The critical requirement for linolenic acid is pollen development, not photosynthesis, in an Arabidopsis mutant. Plant Cell 8: 403–416. 12239389 10.1105/tpc.8.3.403 PMC161109

[pcz201-B51] Melotto M., Underwood W., Koczan J., Nomura K., He S.Y. (2006) Plant stomata function in innate immunity against bacterial invasion. Cell 126: 969–980. 16959575 10.1016/j.cell.2006.06.054

[pcz201-B52] Mitsuda N., Seki M., Shinozaki K., Ohme-Takagi M. (2005) The NAC transcription factors NST1 and NST2 of Arabidopsis regulate secondary wall thickenings and are required for anther dehiscence. Plant Cell 17: 2993–3006. 16214898 10.1105/tpc.105.036004 PMC1276025

[pcz201-B53] Nagpal P., Ellis C.M., Weber H., Ploense S.E., Barkawi L.S., Guilfoyle T.J., et al (2005) Auxin response factors ARF6 and ARF8 promote jasmonic acid production and flower maturation. Development 132: 4107–4118. 16107481 10.1242/dev.01955

[pcz201-B54] Nelson M.R., Band L.R., Dyson R.J., Lessinnes T., Wells D.M., Yang C., et al (2012) A biomechanical model of anther opening reveals the roles of dehydration and secondary thickening. New Phytol. 196: 1030–1037. 22998410 10.1111/j.1469-8137.2012.04329.x PMC3569878

[pcz201-B55] Ogawa M., Kay P., Wilson S., Swain S.M. (2009) ARABIDOPSIS DEHISCENCE ZONE POLYGALACTURONASE1 (ADPG1), ADPG2, and QUARTET2 are polygalacturonases required for cell separation during reproductive development in Arabidopsis. Plant Cell 21: 216–233. 19168715 10.1105/tpc.108.063768 PMC2648098

[pcz201-B56] Park J.H., Halitschke R., Kim H.B., Baldwin I.T., Feldmann K.A., Feyereisen R. (2002) A knock-out mutation in allene oxide synthase results in male sterility and defective wound signal transduction in *Arabidopsis* due to a block in jasmonic acid biosynthesis. Plant J. 31: 1–12. 12100478 10.1046/j.1365-313x.2002.01328.x

[pcz201-B57] Pauwels L., Barbero G.F., Geerinck J., Tilleman S., Grunewald W., Perez A.C., et al (2010) NINJA connects the co-repressor TOPLESS to jasmonate signalling. Nature 464: 788–791. 20360743 10.1038/nature08854 PMC2849182

[pcz201-B58] Peng Y.J., Shih C.F., Yang J.Y., Tan C.M., Hsu W.H., Huang Y.P., et al (2013) A RING-type E3 ligase controls anther dehiscence by activating the jasmonate biosynthetic pathway gene DEFECTIVE IN ANTHER DEHISCENCE1 in Arabidopsis. Plant J. 74: 310–327. 23347376 10.1111/tpj.12122

[pcz201-B59] Prigge M.J., Kadakia N., Greenham K., Estelle M. (2019) Members of the Arabidopsis auxin receptor gene family are essential early in embryogenesis and have broadly overlapping functions, bioRxiv 529248; doi: 10.1101/529248.

[pcz201-B60] Qi T., Huang H., Song S., Xie D. (2015) Regulation of jasmonate-mediated stamen development and seed production by a bHLH-MYB complex in Arabidopsis. Plant Cell 27: 1620–1633. 26002869 10.1105/tpc.15.00116 PMC4498206

[pcz201-B61] Reeves P.H., Ellis C.M., Ploense S.E., Wu M.F., Yadav V., Tholl D., et al (2012) A regulatory network for coordinated flower maturation. PLoS Genet. 8: e1002506. 22346763 10.1371/journal.pgen.1002506 PMC3276552

[pcz201-B62] Rubio-Somoza I., Weigel D. (2013) Coordination of flower maturation by a regulatory circuit of three microRNAs. PLoS Genet. 9: e1003374. 23555288 10.1371/journal.pgen.1003374 PMC3610633

[pcz201-B63] Sanders P.M., Bui A.Q., Le B.H., Goldberg R.B. (2005) Differentiation and degeneration of cells that play a major role in tobacco anther dehiscence. Sex. Plant Reprod. 17: 219–241.

[pcz201-B64] Sanders P.M., Bui A.Q., Weterings K., McIntire K.N., Hsu Y.-C., Lee P.Y., et al (1999) Anther developmental defects in *Arabidopsis thaliana* male-sterile mutants. Sex. Plant Reprod. 11: 297–322.

[pcz201-B65] Sanders P.M., Lee P.Y., Biesgen C., Boone J.D., Beals T.P., Weiler E.W., et al (2000) The Arabidopsis *DELAYED DEHISCENCE1* gene encodes an enzyme in the jasmonic acid synthesis pathway. Plant Cell 12: 1041–1061. 10899973 10.1105/tpc.12.7.1041 PMC149048

[pcz201-B66] Scholz S.S., Reichelt M., Boland W., Mithofer A. (2015) Additional evidence against jasmonate-induced jasmonate induction hypothesis. Plant Sci 239: 9–14. 26398786 10.1016/j.plantsci.2015.06.024

[pcz201-B67] Sheard L.B., Tan X., Mao H., Withers J., Ben-Nissan G., Hinds T.R., et al (2010) Jasmonate perception by inositol-phosphate-potentiated COI1-JAZ co-receptor. Nature 468: 400–405. 20927106 10.1038/nature09430 PMC2988090

[pcz201-B68] Shin B., Choi G., Yi H., Yang S., Cho I., Kim J., et al (2002) AtMYB21, a gene encoding a flower-specific transcription factor, is regulated by COP1. Plant J. 30: 23–32. 11967090 10.1046/j.1365-313x.2002.01264.x

[pcz201-B69] Song S., Chen Y., Liu L., See Y.H.B., Mao C., Gan Y., et al (2018) OsFTIP7 determines auxin-mediated anther dehiscence in rice. Nat Plants 4: 495–504. 29915329 10.1038/s41477-018-0175-0

[pcz201-B70] Song S., Qi T., Huang H., Ren Q., Wu D., Chang C., et al (2011) The Jasmonate-ZIM domain proteins interact with the R2R3-MYB transcription factors MYB21 and MYB24 to affect Jasmonate-regulated stamen development in Arabidopsis. Plant Cell 23: 1000–1013. 21447791 10.1105/tpc.111.083089 PMC3082250

[pcz201-B71] Staswick P.E., Tiryaki I. (2004) The oxylipin signal jasmonic acid is activated by an enzyme that conjugates it to isoleucine in Arabidopsis. Plant Cell 16: 2117–2127. 15258265 10.1105/tpc.104.023549 PMC519202

[pcz201-B72] Steiner-Lange S., Unte U.S., Eckstein L., Yang C., Wilson Z.A., Schmelzer E., et al (2003) Disruption of *Arabidopsis thaliana* MYB26 results in male sterility due to non-dehiscent anthers. Plant J. 34: 519–528. 12753590 10.1046/j.1365-313x.2003.01745.x

[pcz201-B73] Stenzel I., Otto M., Delker C., Kirmse N., Schmidt D., Miersch O., et al (2012) ALLENE OXIDE CYCLASE (AOC) gene family members of *Arabidopsis thaliana*: tissue- and organ-specific promoter activities and in vivo heteromerization. J. Exp. Bot. 63: 6125–6138. 23028017 10.1093/jxb/ers261 PMC3481204

[pcz201-B74] Stintzi A., Browse J. (2000) The *Arabidopsis* male-sterile mutant, *opr3*, lacks the 12-oxophytodienoic acid reductase required for jasmonate synthesis. Proc. Natl. Acad. Sci. U.S.A. 97: 10625–10630. 10973494 10.1073/pnas.190264497 PMC27075

[pcz201-B75] Suza W.P., Staswick P.E. (2008) The role of JAR1 in jasmonoyl-L-isoleucine production during *Arabidopsis* wound response. Planta 227: 1221–1232. 18247047 10.1007/s00425-008-0694-4

[pcz201-B76] Tabata R., Ikezaki M., Fujibe T., Aida M., Tian C.E., Ueno Y., et al (2010) Arabidopsis auxin response factor6 and 8 regulate jasmonic acid biosynthesis and floral organ development via repression of class 1 KNOX genes. Plant Cell Physiol. 51: 164–175. 20007966 10.1093/pcp/pcp176

[pcz201-B77] Tashiro S., Tian C.E., Watahiki M.K., Yamamoto K.T. (2009) Changes in growth kinetics of stamen filaments cause inefficient pollination in massugu2, an auxin insensitive, dominant mutant of *Arabidopsis thaliana*. Physiol. Plant. 137: 175–187. 19719484 10.1111/j.1399-3054.2009.01271.x

[pcz201-B78] Tatematsu K., Kumagai S., Muto H., Sato A., Watahiki M.K., Harper R.M., et al (2004) MASSUGU2 encodes Aux/IAA19, an auxin-regulated protein that functions together with the transcriptional activator NPH4/ARF7 to regulate differential growth responses of hypocotyl and formation of lateral roots in *Arabidopsis thaliana*. Plant Cell 16: 379–393. 14729917 10.1105/tpc.018630 PMC341911

[pcz201-B79] Thines B., Katsir L., Melotto M., Niu Y., Mandaokar A., Liu G., et al (2007) JAZ repressor proteins are targets of the SCF^COI1^ complex during jasmonate signalling. Nature 448: 661–665. 17637677 10.1038/nature05960

[pcz201-B80] Wasternack C. (2007) Jasmonates: an update on biosynthesis, signal transduction and action in plant stress response, growth and development. Ann. Bot. 100: 681–697. 17513307 10.1093/aob/mcm079 PMC2749622

[pcz201-B81] Wei D., Liu M., Chen H., Zheng Y., Liu Y., Wang X., et al (2018) INDUCER OF CBF EXPRESSION 1 is a male fertility regulator impacting anther dehydration in Arabidopsis. PLoS Genet. 14: e1007695. 30286083 10.1371/journal.pgen.1007695 PMC6191155

[pcz201-B82] Wilson Z.A., Song J., Taylor B., Yang C. (2011) The final split: the regulation of anther dehiscence. J. Exp. Bot. 62: 1633–1649. 21325605 10.1093/jxb/err014

[pcz201-B83] Wu M.F., Tian Q., Reed J.W. (2006) Arabidopsis microRNA167 controls patterns of ARF6 and ARF8 expression, and regulates both female and male reproduction. Development 133: 4211–4218. 17021043 10.1242/dev.02602

[pcz201-B84] Xie D.X., Feys B.F., James S., Nieto-Rostro M., Turner J.G. (1998) *COI1*: an *Arabidopsis* gene required for jasmonate-regulated defense and fertility. Science 280: 1091–1094. 9582125 10.1126/science.280.5366.1091

[pcz201-B85] Yan J., Li S., Gu M., Yao R., Li Y., Chen J., et al (2016) Endogenous bioactive jasmonate is composed of a set of (+)-7-iso-JA-amino acid conjugates. Plant Physiol. 172: 2154–2164. 27756820 10.1104/pp.16.00906 PMC5129707

[pcz201-B86] Yang C., Song J., Ferguson A.C., Klisch D., Simpson K., Mo R., et al (2017) Transcription factor MYB26 is key to spatial specificity in anther secondary thickening formation. Plant Physiol. 175: 333–350. 28724622 10.1104/pp.17.00719 PMC5580765

[pcz201-B87] Yang C., Xu Z., Song J., Conner K., Vizcay Barrena G., Wilson Z.A. (2007) Arabidopsis MYB26/MALE STERILE35 regulates secondary thickening in the endothecium and is essential for anther dehiscence. Plant Cell 19: 534–548. 17329564 10.1105/tpc.106.046391 PMC1867336

[pcz201-B88] Yang X.Y., Li J.G., Pei M., Gu H., Chen Z.L., Qu L.J. (2007) Over-expression of a flower-specific transcription factor gene AtMYB24 causes aberrant anther development. Plant Cell Rep. 26: 219–228. 16972096 10.1007/s00299-006-0229-z

[pcz201-B89] Zhao Z., Zhang Y., Liu X., Zhang X., Liu S., Yu X., et al (2013) A role for a dioxygenase in auxin metabolism and reproductive development in rice. Dev Cell 27: 113–122. 24094741 10.1016/j.devcel.2013.09.005

[pcz201-B90] Zheng L., Nagpal P., Villarino G., Trinidad B., Bird L., Huang Y., et al (2019) miR167 limits anther growth to potentiate anther dehiscence. Development 146: dev174375; doi: 10.1242/dev.174375 10.1242/dev.174375 31262724

